# Sport participation after unicompartmental knee arthroplasty: High return rates independent of implant design or technique, a systematic review and meta‐analysis

**DOI:** 10.1002/jeo2.70514

**Published:** 2026-01-11

**Authors:** Tosca Cerasoli, Antongiulio Favero, Federico Coliva, Alberto Fogacci, Andrea Pasquini, Stefano Zaffagnini, Giulio Maria Marcheggiani Muccioli

**Affiliations:** ^1^ II Clinica Ortopedica – IRCCS Istituto Ortopedico Rizzoli Bologna Italy; ^2^ UNIBO‐University of Bologna Bologna Italy; ^3^ Department of Orthopaedics and Traumatology “Pius Brinzeu” Emergency Clinical County Hospital Timisoara Romania

**Keywords:** return to sport after knee arthroplasty, RTS, sport, unicompartmental knee arthroplasty

## Abstract

**Purpose:**

Unicompartmental knee arthroplasty (UKA) is a widely used treatment for isolated compartmental knee osteoarthritis, especially in younger and more active patients. Return to sport (RTS) has become a key postoperative outcome. While RTS after UKA is generally favourable, the influence of factors such as implant design, surgical technique, compartment treated and patient characteristics remains unclear. An updated and granular synthesis is needed, particularly to allow direct comparison with RTS outcomes after total knee arthroplasty (TKA).

**Methods:**

A systematic review and meta‐analysis was conducted following PRISMA guidelines and registered on PROSPERO. PubMed, Embase and Scopus were searched for studies from 2016 to April 2025 reporting RTS outcomes after UKA. Two reviewers independently screened articles, extracted data and assessed methodological quality using the modified Coleman Methodology Score. Weighted means and proportions were calculated, and correlation analyses explored associations between RTS and patient and surgical variables.

**Results:**

Thirteen studies (*n* = 1675 patients) were included. The overall weighted mean RTS rate after UKA was 87.97% with a substantial proportion of patients resuming intermediate and high‐impact sports. No significant differences in RTS were found between fixed‐bearing and mobile‐bearing implants, or between medial and lateral UKA. RTS was comparable between robotic‐assisted and standard techniques. A moderate negative correlation between BMI and RTS was observed (*r* = −0.69, *p* = 0.019). Compared to TKA, UKA patients have a higher RTS rate (87.97% vs. 72%). UKA patients resumed a broader range of sports, with increased participation in intermediate‐ and high‐impact activities.

**Conclusions:**

UKA offers high RTS rates and supports participation in demanding physical activities. Neither implant design nor surgical technique substantially impacts RTS, whereas patient‐related factors, especially BMI, are key predictors. Compared to TKA, UKA is associated with superior sport‐related outcomes, underscoring its role in appropriately selected patients. These findings support shared decision‐making and personalised postoperative planning.

**Level of Evidence:**

Level III.

**Abbreviations**
BMI: body mass index
CI: confidence interval
FB‐MB: fixed bearing‐metal backed
LUKA: lateral unicompartmental knee arthroplasty
MB‐MB: mobile bearing‐metal backed
MUKA: medial unicompartmental knee arthroplasty
NRTS: no return to sport
PRISMA: preferred reporting items for systematic reviews and meta‐analyses
RTS: return to sport
SD: standard deviation
TKA: total knee arthroplasty
UCLA: University of California at Los Angeles – Activity Score
UKA: unicompartmental knee arthroplasty
VAS: visual analogue scale

## INTRODUCTION

Unicompartmental knee arthroplasty (UKA) is a well‐established surgical option for the treatment of isolated compartmental knee osteoarthritis, particularly in younger and more active patients. Compared to total knee arthroplasty (TKA), UKA offers numerous potential advantages, including faster recovery, better preservation of native knee kinematics and improved functional outcomes in selected patients [30, 33]. Among these functional outcomes, the ability to return to sport (RTS) has gained increasing importance in recent years, both for patients and surgeons. The opportunity to resume physical activity is often a key expectation for patients undergoing UKA, and it can substantially influence postoperative satisfaction and quality of life [17].

A growing number of studies have investigated RTS after UKA, reporting generally favourable results. However, there is substantial heterogeneity in the literature regarding how RTS is defined and measured, and in which factors may influence the likelihood of returning to sport. Two recent systematic reviews and meta‐analyses, published in 2022 and 2024, have summarised RTS outcomes after UKA, providing valuable insights into this topic [30, 33]. Both analyses demonstrated high RTS rates and generally good functional outcomes. However, the reviews primarily focused on overall RTS, without systematically analysing the influence of other potentially relevant variables such as patient characteristics (age, body mass index [BMI]), the specific compartment treated (medial vs. lateral UKA), or implant design (fixed‐bearing metal‐backed [FB‐MB] vs. mobile‐bearing metal‐backed [MB‐MB]). Moreover, since these publications, additional studies have become available, further enriching the current literature and warranting an updated and more granular analysis.

Importantly, in parallel with the growing interest in RTS after knee arthroplasty, several large and contemporary series have clarified RTS outcomes after TKA and UKA [20]. This underscores the need for updated and comparable data on RTS after UKA to better inform clinical decision‐making and patient counselling. Furthermore, in order to enable a robust and methodologically consistent comparison between UKA and TKA, we adopted a unified literature search strategy covering all recent studies on RTS following knee arthroplasty. This approach allowed us to develop two companion systematic reviews and meta‐analyses, one on UKA (the present study) and one on TKA, designed to provide directly comparable results regarding RTS outcomes in these two surgical contexts [6].

In this framework, we conducted an updated systematic review and meta‐analysis to provide a comprehensive synthesis of the current literature on RTS after UKA. Our aim was to identify and analyse the key factors that may influence RTS outcomes and to provide a detailed analysis of the types of sport resumed after UKA, offering updated evidence to support both surgeons and patients in the shared decision‐making process.

## MATERIALS AND METHODS

**Figure 1:**
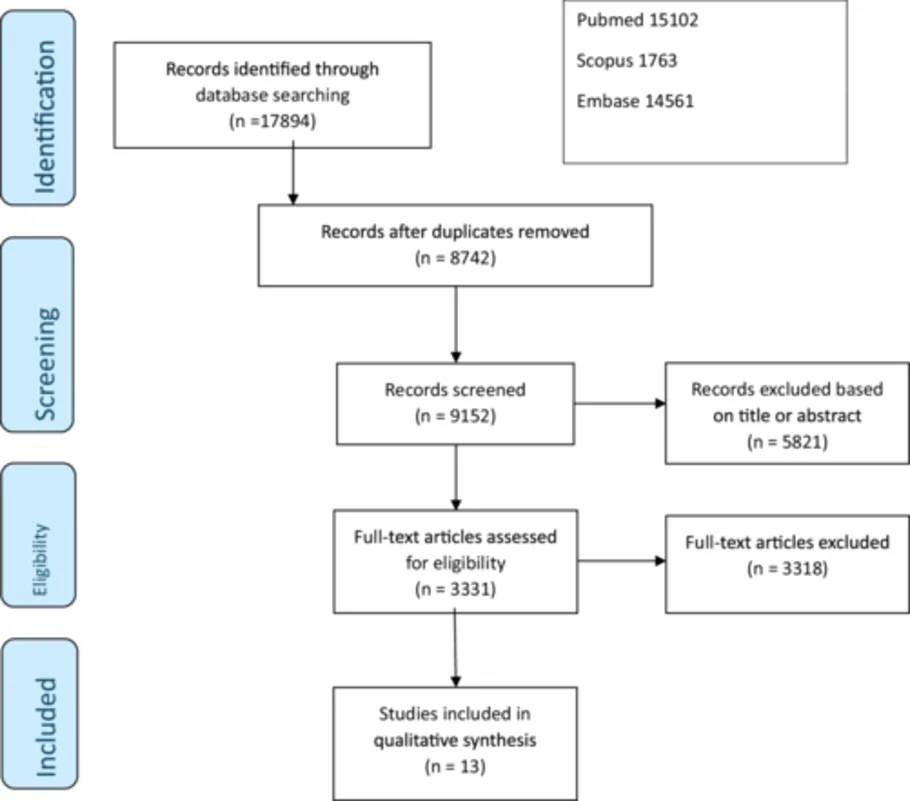
PRISMA 2009 flow diagram.

This systematic review and meta‐analysis were conducted in accordance with the PRISMA guidelines and were prospectively registered in the PROSPERO database (CRD420251032511) (Figure 1). The aim of the study was to investigate the determinants of RTS after UKA, including patient‐related factors, surgical factors and implant characteristics.

A systematic literature search was performed in PubMed, Embase and Scopus for studies published between 1 January 2016, and 30 April 2025. The complete search strategy was as follow: (knee arthroplasty OR knee replacement OR TKA OR UKA) AND (RTS OR physical activity OR return to activity OR athletic participation) AND (implant design OR fixed bearing OR mobile bearing OR medial pivot OR posterior stabilised OR cruciate retaining). The reference lists of included articles and relevant reviews were also screened to identify additional eligible studies.

The present review was conducted in parallel with a companion systematic review on RTS after TKA, using a unified and broad search strategy to allow for consistent comparison across the two arthroplasty types.

Two reviewers independently screened titles and abstracts and performed full‐text review of potentially eligible articles. Disagreements were resolved through discussion with a third reviewer. Studies were included if they reported RTS outcomes in patients treated with UKA. Studies were excluded if they did not report RTS outcomes, if they included patients undergoing TKA, revision UKA, or multiple surgical procedures performed simultaneously, or if they included patients with severe concomitant conditions potentially influencing physical function, such as neurological or systemic diseases.

Data extraction was performed independently by two reviewers using a standardised form. The following data were extracted: study characteristics (first author, year of publication, study design, number of patients, mean follow‐up), patient characteristics (age, BMI), RTS outcomes (RTS rate, type of sport resumed, time to RTS), physical activity scores (Tegner, UCLA) and additional factors potentially influencing RTS (implant type when reported, medial vs. lateral UKA, patient satisfaction, reasons for not returning to sport and time for RTS).

The methodological quality of the included studies was assessed using the modified coleman methodology score (mCMS). Two reviewers independently applied the scoring system and resolved discrepancies through consensus.

### Statistical analysis

Descriptive statistics were used to summarise patient demographics and study characteristics. For dichotomous outcomes, such as RTS rate, weighted mean proportions were calculated. For continuous outcomes, including age, BMI, follow‐up duration, Tegner and UCLA activity scores, weighted means and weighted standard deviations were computed.

The association between age, BMI and follow‐up duration with RTS rate was assessed using Pearson correlation coefficients. Additional correlation analyses were performed to explore potential relationships between RTS rate and physical activity scores. Comparisons of RTS rate and activity scores were also conducted according to implant design (fixed‐bearing vs. mobile‐bearing) and compartment treated (medial vs. lateral UKA), when reported.

In addition, RTS in the UKA cohort were compared to those reported in a companion systematic review on TKA [6] using independent‐samples *t*‐tests. Statistical significance was set at *p* < 0.05.

Given the expected heterogeneity among studies, a random‐effects model was used for meta‐analyses. Statistical heterogeneity was assessed using the *I*² statistic and chi‐squared test. All statistical analyses were performed using Python (SciPy and Statsmodels packages) and Excel.

## RESULTS

**Figure 2:**
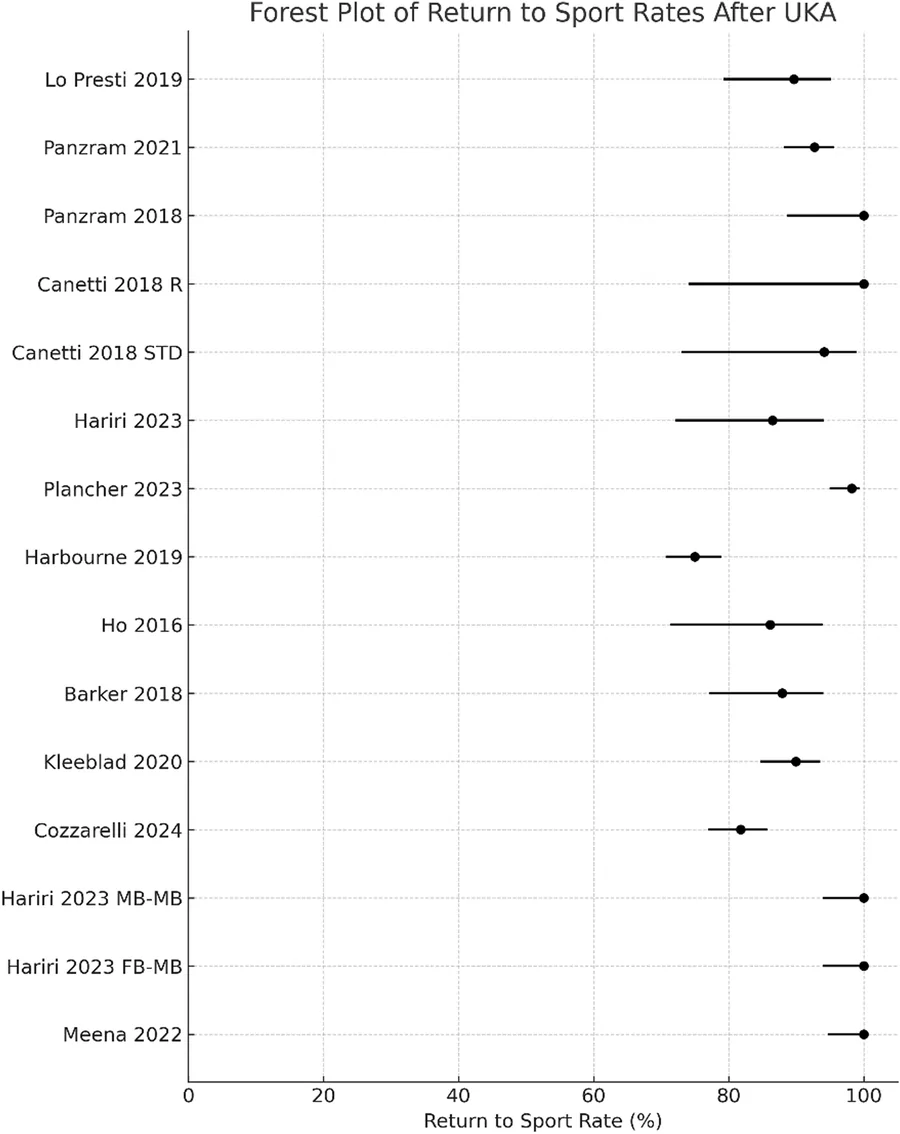
Forest plot of return to sport (RTS) rates after unicompartmental knee arthroplasty (UKA). The diagram shows the RTS rates after UKA across 15 study arms derived from 13 included studies. Two studies reported separate RTS rates (Canetti 2018 one arm using standard technique and one arm using robotic technique [5], Hariri 2023 one arm for mobile bearing‐metal backed [MB‐MB] and one arm for fixed bearing‐metal backed [FB‐MB] [13]), resulting in a total of 15 data points. Each point represents the proportion of patients who resumed sport activities postoperatively, with 95% confidence intervals computed using the Wilson method.

Thirteen studies were included in the final analysis, for a total of 1675 patients. The weighted mean patient age was 63.5 years (SD 10.1), mean BMI was 28.5 kg/m² (SD 6.4) and mean follow‐up was 32.6 months (SD 18.1). The overall weighted mean RTS rate was 87.2% (the included studies are reported in Table 1 and in Figure 2).

**Table 1:** Summary of the included articles.

Reference	Number of implants	Age (in years)	Age DS (in years)	BMI	BMI DS	Type of implant	Follow‐up (in months)	Follow‐up DS (in months)
[22]	58	59.7	5.0			MUKA FB‐MB	48	6
[29]	192					UKA MB‐MB	24	
[28]	30	62.5	6.6			MUKA MB‐MB	60	8.3
[5]	11	66.5	6.8	24.2	4.3	LUKA FB‐AP	34.4	10.5
[5]	17	59.5	9.9	26.3	3.8	LUKA FB‐AP	39.3	15.5
[14]	37	52.8	3.1	30	6.8	LUKA	37	18
[31]	169	59.6	5	27.6	4	UKA	60	
[12]	420	67	10	30	5	UKA	12	
[16]	36	60	15.3	30.3	26.0	UKA	45.6	10.9
[1]	58	68.9	9.2			UKA	12	
[18]	179	62.3	8.8	27.6	4.4	UKA FB‐MB	20.2	
[7]	296	61.3	10.0	28.2	4.2	UKA	55.2	
[15]	60	61.3	10.3	27.8	5.6	LUKA MB‐MB	40.8	15.6
[15]	60	61.4	11.2	27.5	6	LUKA FB‐MB	32.4	14.4
[24]	69	74.2	8.8	26.8	3.2	UKA MB‐MB	24	

Abbreviations: BMI, body mass index; DS, standard deviation; FB‐MB, fixed bearing metal backed; LUKA, lateral UKA; MB‐MB, mobile bearing metal backed; MUKA, medial UKA; R, robot; STD, standard; UKA, unicompartmental knee arthroplasty.

Four studies included UKA arms from mixed cohorts comparing UKA and TKA [1, 12, 16, 24], while two studies had two arms comparing either different implant designs or surgical techniques [5, 15]. The study by Canetti et al evaluated RTS intensity, reporting that patients undergoing robotic‐assisted UKA returned to sport at the same intensity more frequently than those treated with conventional techniques (91% vs. 82.4%) [5]. The study by Hariri et al. compared two different prosthetic designs FB‐MB and MB‐MB reporting a statistically significant difference in favour of FB‐MB [15]. The study by Meena et al. reported only the frequency of sport participation without specifying sport type or intensity [24]. Similarly, the study by Ho et al. assessed patients’ satisfaction regarding their ability to perform sport compared to the preoperative period, with 74% of patients reporting being satisfied or improved [16]. The study by Barker et al. reports only the type of sport resumed postoperatively and the dissatisfaction among the resumed sports (17% swimming, 31% cycling, 10% other) without reporting the reasons [1]. Finally, one study focused exclusively on low‐impact sports [15].

The included articles were published between 2016 and 2024. Five studies reported results for medial or lateral UKA separately [5, 14, 15, 22, 28]. Three studies compared FB‐MB and MB‐MB without specifying medial or lateral compartment [18, 24, 29]. Five studies reported RTS outcomes generically for UKA [1, 2, 7, 12, 16]. Robotic‐assisted technique was reported in only two studies [5, 18], while all other studies employed standard surgical techniques; in two studies the surgical technique was not specified [1, 7].

### Sports

**Figure 3:**
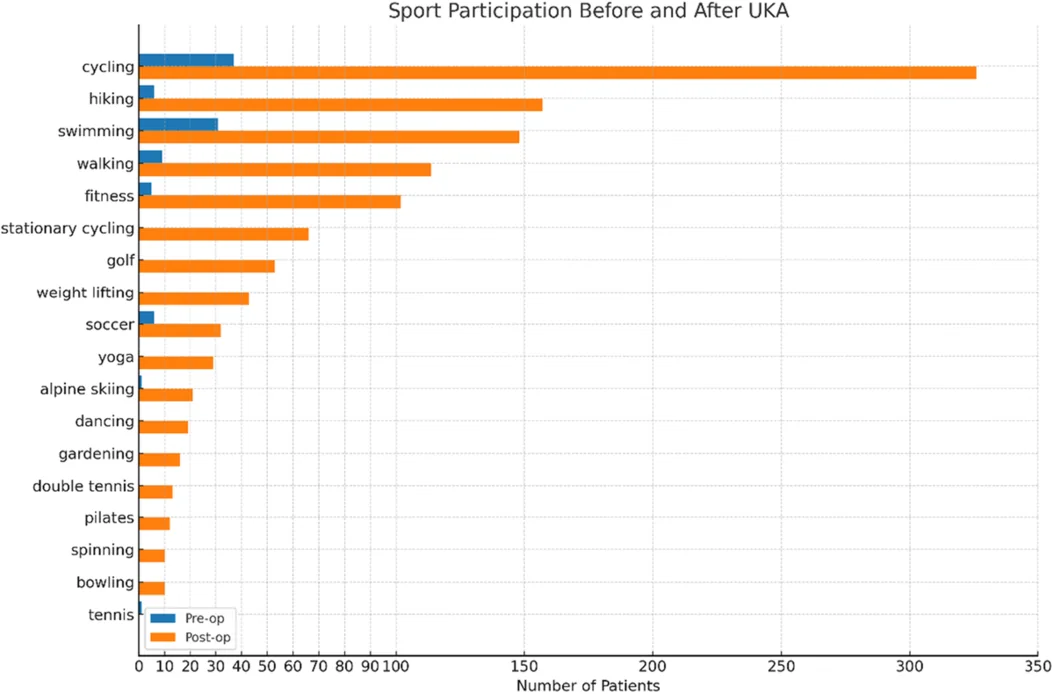
Sport participation before and after unicompartmental knee arthroplasty (UKA). Bar chart illustrating the number of patients participating in various sports preoperatively (blue) and postoperatively (orange). A nonlinear scale was applied to the *x*‐axis to enhance visual resolution for the lower range (0–100).

The type of sport practiced before and after surgery was inconsistently reported across the included studies. In many cases, the type of sport was only detailed postoperatively, which may partly explain the apparent increase in participation in intermediate‐ and high‐impact sports after UKA. The specific types of sport practiced pre‐ and postoperatively are depicted in Figure 3.

**Figure 4:**
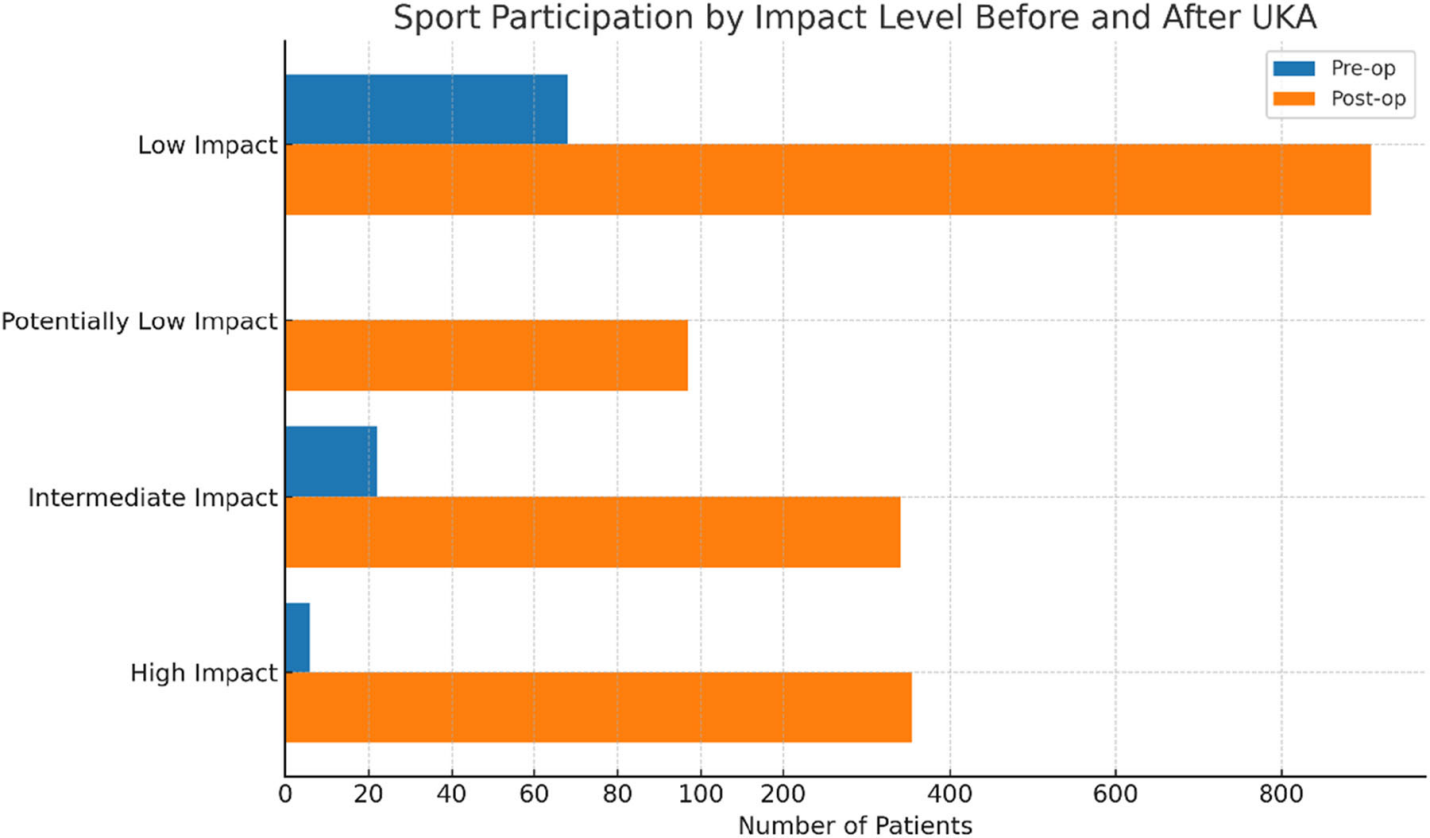
Sport participation by impact level before and after unicompartmental knee arthroplasty (UKA). Bar chart showing the number of patients engaged in low‐, intermediate‐, and high‐impact sports preoperatively (blue) and postoperatively (orange). A nonlinear scale was applied to the *x*‐axis to enhance visual resolution for the lower range (0–100).

To enable the inclusion of studies that did not report individual sports but rather categorised activity level by impact (low, intermediate and high), we applied the classification proposed by Vail et al. [35]. This approach allowed for a more uniform comparison of sport participation across studies with heterogeneous reporting. The number of patients practicing sports in each impact category before and after surgery, as derived from this standardised classification, is also presented in Figure 4. It should be noted that the total number of reported sports exceeds the total number of patients, as many patients participated in more than one sport.

### Sport participation pattern UKA versus TKA

Sport participation patterns after TKA, as reported in a companion systematic review conducted with the same search strategy and methodology [6] showed a predominant shift towards low‐impact activities. Cycling, swimming and skiing were the most frequently practiced sports after TKA. Participation in running decreased from 18.4% preoperatively to 8.5% postoperatively, while skiing participation remained relatively stable (22%–20%). The proportion of intermediate‐impact sports decreased from 20.8% preoperatively to 15.0% postoperatively, and high‐impact sports decreased from 17.8% to 14.1%. Low‐impact sports remained predominant, accounting for 58.8% of preoperative and 56.9% of postoperative participation.

The UKA cohort demonstrated a diversified pattern of sport participation postoperatively, extending beyond low‐impact disciplines. Low‐impact sports remained the most commonly reported category (51.8%), although their relative proportion decreased compared to the preoperative period (70.8%). Participation in intermediate‐impact sports remained relatively stable (22.9% preoperatively vs. 20.4% postoperatively), while high‐impact sports increased from 6.3% to 21.1%. Postoperatively, participation was also reported in several sports generally classified as high‐demanding such as alpine skiing (1.8%), double tennis (1.1%), soccer (2.7%) and weight lifting (3.7%).

It should be noted that the number of sports reported postoperatively was substantially higher than in the preoperative period, which may have led to an underrepresentation of preoperative participation.

## META‐ANALYSIS

### Demographics

Correlation analyses explored the influence of key demographic and study‐level factors on RTS. A negative association was found between study mean BMI and RTS rate (Pearson *r* = –0.69; *p* = 0.019), indicating that cohorts with higher average BMI tended to report lower RTS rates. In contrast, mean patient age showed no significant relationship with RTS (*r* = 0.10; *p* = 0.72), suggesting age did not materially affect the likelihood of RTS. Finally, follow‐up duration was weakly positively correlated with RTS (*r* = 0.29), but this was not statistically significant (*p* = 0.30), suggesting that the length of follow‐up did not substantially influence reported RTS rates. This supports the reliability of RTS data even from studies with shorter follow‐up periods.

### FB‐MB versus MB‐MB

Additional analyses compared outcomes between FB‐MB and MB‐MB implants. RTS rate did not significantly differ between FB‐MB (92.4%) and MB‐MB (95.2%) groups (*p* = 0.19). Similarly, no significant difference was found in postoperative VAS scores (FB‐MB: 1.3 ± 1.7; MB‐MB: 1.4 ± 2.5; *p* = 0.55).

Analysis of UCLA activity level revealed significant differences between groups. Preoperative UCLA scores were significantly higher in FB‐MB patients compared to MB‐MB patients (5.26 ± 3.51 vs. 2.82 ± 1.66; *p* < 0.001). Postoperative UCLA scores remained significantly higher in the FB‐MB group (6.55 ± 1.84 vs. 6.15 ± 1.45; *p* = 0.003). However, the improvement in activity level (ΔUCLA) was significantly greater in MB‐MB patients (3.45 ± 0.14) compared to FB‐MB patients (1.48 ± 1.47; *p* < 0.001), indicating a more pronounced postoperative gain in activity among MB‐MB patients.

Postoperative Tegner activity level was reported in one FB‐MB study (Tegner post 3.10 ± 1.20) and in four MB‐MB studies (weighted mean Tegner post 3.50 ± 1.30). Given the limited number of FB‐MB studies available, no formal statistical comparison was performed.

### LUKA versus MUKA

Finally, outcomes were compared between medial and lateral UKA. RTS rate was slightly higher in LUKA patients (97%) compared to MUKA patients (93.4%), though the difference was not statistically significant (*p* = 0.21). Postoperative VAS was lower in MUKA patients (1.1 ± 0.6) compared to LUKA patients (2.1 ± 2.5), although no formal statistical comparison was performed due to limited data.

UCLA activity level was available preoperatively in three of five LUKA studies (weighted mean UCLA pre 2.47 ± 2.61) and postoperatively in all LUKA studies (UCLA post 5.94 ± 1.54). For MUKA, postoperative UCLA was reported in one study only (6.10 ± 1.80), while preoperative UCLA data were not provided. Given the heterogeneity of available data, no formal statistical comparison was performed between LUKA and MUKA groups.

Postoperative Tegner activity level was reported in two LUKA studies (weighted mean Tegner post 3.05 ± 1.11) and in one MUKA study (3.40 ± 1.00). Again, no formal statistical comparison was performed due to limited data and heterogeneity.

### Standard technique versus robotic technique

In total, 1148 patients treated using a standard surgical technique and 190 patients treated with a robotic‐assisted technique were included in the analysis. The weighted mean RTS rate was 87.97% in the standard technique group and 90.59% in the robotic group. The difference in RTS rate between the two groups was not statistically significant (*p* = 0.192). The absolute risk difference (RD) between robotic and standard technique was +2.6% (95% confidence interval [CI]: −0.9% to +6.1%), while the relative risk (RR) of returning to sport with robotic versus standard technique was 1.03 (95% CI: 0.99–1.07). Overall, while a trend towards higher RTS rates was observed in the robotic group, this difference did not reach statistical significance.

### TKA versus UKA

Patient age and BMI were compared between the present UKA cohort and the TKA cohort analysed in a companion systematic review conducted using the same search strategy and methodology [6].

In the TKA cohort (*n* = 3795), the mean age was 66.95 ± 9.44 years and the mean BMI was 29.62 ± 8.42 kg/m². In comparison, patients included in the UKA studies were significantly younger (63.5 ± 10.1 years, *n* = 1675; *p* < 0.00001) and had a significantly lower BMI (28.5 ± 6.4 kg/m²; *p* < 0.000001).

Moreover, the overall RTS rate was substantially higher in UKA (87.97%) than in TKA (72%), further confirming the more favourable sport‐related outcomes typically observed after UKA.

## DISCUSSION

This updated systematic review and meta‐analysis confirms that UKA enables high RTS rates in patients with isolated compartmental osteoarthritis. Our overall weighted mean RTS rate of 87.97% is consistent with the broader UKA literature [2, 20, 30] and, in particular, aligns with the most recent meta‐analysis by Radhakrishnan et al., which reported a pooled RTS rate of 92.7%, further underscoring the reliability of UKA in restoring physical activity [33].

We sought to explore which factors may influence RTS after UKA.

Our analysis highlights that neither implant design (FB‐MB vs. MB‐MB) nor compartment treated (medial vs. lateral) significantly influenced RTS rates. While previous literature has demonstrated excellent implant survivorship for both designs, with some studies suggesting superior long‐term survival for MB‐MB in medial UKA and for FB‐MB implants in general [10, 11], these differences appear to be less relevant when considering short‐ to mid‐term functional outcomes such as RTS. Our results suggest that RTS is primarily driven by patient‐related factors rather than by implant characteristics or compartment treated.

Similarly, RTS was comparable between standard and robotic‐assisted UKA. Although a trend toward higher RTS was observed in robotic cases, this difference did not reach statistical significance, confirming findings from recent reports suggesting that robotic assistance may improve implant positioning but does not necessarily translate into superior short‐term functional outcomes [5, 12, 33].

We observed a moderate negative correlation between BMI and RTS, indicating that higher BMI may limit the likelihood of sport resumption after UKA. This finding is consistent with the data from TKA cohorts, where elevated BMI has been associated with lower functional outcomes and reduced physical activity levels [25, 27]. While UKA was once considered less suitable for patients with elevated BMI, recent studies have reported favourable outcomes and survivorship in this population [4]. However, these studies mainly focused on implant survival and general clinical outcomes rather than on RTS. In particular, one study reported that sports‐related scores improve in patients who lose weight after UKA, although the types of sport were not detailed [8]. This supports the trend observed in our analysis and aligns with broader literature indicating that BMI negatively influences the resumption of physical activity after knee arthroplasty.

In parallel, we compared our results with those from a companion systematic review on TKA conducted using the same search strategy and methodology [6]. This comparison revealed that UKA patients were significantly younger and had a lower BMI than TKA patients, factors that are consistently associated with improved RTS outcomes [1, 7, 9, 34]. Notably, the overall RTS rate was substantially higher in UKA (87.97%) compared to TKA (72%), in line with previous comparative studies [12, 18, 21, 26, 32]. These findings reflect inherent differences in patient selection and likely contribute to the superior RTS outcomes observed in the UKA population.

The analysis of sport participation patterns revealed that UKA patients resumed a wide range of activities, with substantial increases in intermediate and high‐impact sports. Cycling, hiking, swimming and walking were the most commonly practiced sports postoperatively, while activities such as weight lifting, golf and yoga were newly introduced after surgery. These findings support the notion that UKA preserves the functional capacity required for more demanding activities [22, 29].

Sport participation patterns also differed when compared to TKA [6]. While UKA patients demonstrated increase engagement in intermediate and high‐impact activities, TKA patients showed a shift toward lower‐impact sports. Notably, skiing remained a common activity in both groups, reflecting the growing confidence of surgeons and patients in resuming demanding sports after arthroplasty when appropriate patient selection and rehabilitation are applied [3, 19, 23, 36]. However, this finding may also reflect a selection bias, as sports traditionally discouraged after knee arthroplasty such as skiing and golf tend to receive disproportionate attention in the literature.

The present findings must be interpreted considering several limitations. First, heterogeneity in RTS definitions and reporting persists across studies. Many included studies lacked detailed preoperative sport data, limiting the ability to quantify net changes in sport participation. Additionally, reasons for non‐RTS were inconsistently reported in the UKA literature, representing an area for future research. Another important limitation is the absence of information regarding rehabilitation protocols, which are known to play a critical role in influencing RTS outcomes. These limitations are reflected in the methodological quality of the included studies, with mCMS ranging from 60 to 70, indicative of acceptable but not excellent quality.

Despite these limitations, this study provides an updated and granular synthesis of RTS after UKA, with direct methodological comparability to TKA data. Our results support the view that UKA enables superior sport‐related outcomes compared to TKA and confirm that neither implant design nor surgical technique currently exerts a major influence on RTS rates. Importantly, patient factors, particularly BMI and baseline activity level, remain key determinants of postoperative sport participation. These findings are clinically meaningful, as they can support tailored preoperative counselling and guide individualised rehabilitation strategies based on patient‐specific characteristics and expectations.

## CONCLUSIONS

UKA enables a high rate of RTS, including participation in intermediate‐ and high‐impact activities. Compared to TKA, UKA offers superior sport‐related outcomes, particularly in younger and active patients. Implant design, compartment treated and surgical technique do not appear to substantially influence RTS rates. Patient‐related factors, especially BMI, remain critical predictors. These findings can support shared decision‐making and highlight the importance of personalised rehabilitation strategies to optimise postoperative outcomes.

## AUTHOR CONTRIBUTIONS

All authors contributed to study conception and design. Literature search and data extraction were performed by Federico Coliva and Antongiulio Favero and Alberto Fogacci. Statistical analysis was conducted by Tosca Cerasoli. Tosca Cerasoli drafted the manuscript; all authors critically revised the work for important intellectual content and approved the final version.

## CONFLICT OF INTEREST STATEMENT

The authors declare no conflicts of interest.

## ETHICS STATEMENT

systematic review of published data; no new human or animal subjects were involved.

## Supporting information

Table 1: Modified Coleman Methodology Scores (mCMS) for the studies included in the meta‐analysis on return to sport after UKA. The mCMS was used to assess the methodological quality of each study across ten domains, including study design, sample size, follow‐up, surgical and rehabilitation details, and outcome reporting. Most studies received moderate scores, reflecting common limitations in retrospective designs, incomplete reporting of rehabilitation protocols, and insufficient detail on patient recruitment and selection processes.
Table 2: Number of patients and weighted percentages of sports participation before and after surgery.
Table 3: Number of patients and weighted percentages of sports participation before and after surgery, categorized according to the Vail classification.

## Data Availability

All data used are publicly available in the original published studies included in the meta‐analysis.
