# Supplementary material for: Sport participation after unicompartmental knee arthroplasty: High return rates independent of implant design or technique, a systematic review and meta‐analysis
Source: J Exp Orthop. 2026 Jan 11;13(1):e70514. doi: 10.1002/jeo2.70514 (PMC12793043; doi:10.1002/jeo2.70514)
Supplement: Supplementary file 1 — Table 1: Modified Coleman Methodology Scores (mCMS) for the studies included in the meta‐analysis on return to sport after UKA. The mCMS was used to assess the methodological quality of each study across ten domains, including study design, sample size, follow‐up, surgical and rehabilitation details, and outcome reporting. Most studies received moderate scores, reflecting common limitations in retrospective designs, incomplete reporting of rehabilitation protocols, and insufficient detail on patient recruitment and selection processes. Table 2: Number of patients and weighted percentages of sports participation before and after surgery. Table 3: Number of patients and weighted percentages of sports participation before and after surgery, categorized according to the Vail classification. [file JEO2-13-e70514-s001.docx]

Supplementary material

| **Study** | **Year** | **mCMS Score** |
| --- | --- | --- |
| Lo Presti 2019 | 2019 | 61 |
| Panzram 2021 | 2021 | 70 |
| Panzram 2018 | 2018 | 65 |
| Canetti 2018 robot | 2018 | 66 |
| Canetti 2018 standard | 2018 | 66 |
| Hariri 2023 | 2023 | 63 |
| Plancher 2023 | 2023 | 63 |
| Harbourne 2019 | 2019 | 60 |
| Ho 2016 | 2016 | 63 |
| Barker 2018 | 2018 | 69 |
| Kleeblad 2020 | 2020 | 60 |
| Cozzarelli 2024 | 2024 | 67 |
| Hariri 2023 MB-MB | 2023 | 70 |
| Hariri 2023 FB-MB | 2023 | 70 |
| Meena 2022 | 2022 | 69 |

Table 1: Modified Coleman Methodology Scores (mCMS) for the studies included in the meta-analysis on return to sport after UKA. The mCMS was used to assess the methodological quality of each study across ten domains, including study design, sample size, follow-up, surgical and rehabilitation details, and outcome reporting. Most studies received moderate scores, reflecting common limitations in retrospective designs, incomplete reporting of rehabilitation protocols, and insufficient detail on patient recruitment and selection processes.

Table 2

| **SPORT** | **n° PRE-OP** | **n° POST-OP** | **% PRE-OP** | **% POST-OP** |
| --- | --- | --- | --- | --- |
| cycling | 37 | 326 | 38.54% | 27.84% |
| hiking | 6 | 157 | 6.25% | 13.41% |
| swimming | 31 | 148 | 32.29% | 12.64% |
| walking | 9 | 114 | 9.38% | 9.74% |
| fitness | 5 | 102 | 5.21% | 8.71% |
| stationary cycling | 0 | 66 | 0.00% | 5.63% |
| golf | 0 | 53 | 0.00% | 4.53% |
| weight lifting | 0 | 43 | 0.00% | 3.67% |
| soccer | 6 | 32 | 6.25% | 2.73% |
| yoga | 0 | 29 | 0.00% | 2.48% |
| alpine skiing | 1 | 21 | 1.04% | 1.79% |
| dancing | 0 | 19 | 0.00% | 1.62% |
| gardening | 0 | 16 | 0.00% | 1.37% |
| double tennis | 0 | 13 | 0.00% | 1.11% |
| pilates | 0 | 12 | 0.00% | 1.02% |
| spinning | 0 | 10 | 0.00% | 0.85% |
| bowling | 0 | 10 | 0.00% | 0.85% |
| tennis | 1 | 0 | 1.04% | 0.00% |

Table 2: Number of patients and weighted percentages of sports participation before and after surgery.

Table 3

| **SPORT** | **n° PRE-OP** | **n° POST-OP** | **% PRE-OP** | **% POST-OP** |
| --- | --- | --- | --- | --- |
| low impact | 68 | 746 | 70.83% | 51.81% |
| potentially low impact | 0 | 97 | 0.00% | 6.74% |
| intermediate impact | 22 | 293 | 22.92% | 20.35% |
| high impact | 6 | 304 | 6.25% | 21.11% |

Table 3: Number of patients and weighted percentages of sports participation before and after surgery, categorized according to the Vail classification.
